# The Role of the Cullin-5 E3 Ubiquitin Ligase in the Regulation of Insulin Receptor Substrate-1

**DOI:** 10.1155/2012/282648

**Published:** 2012-12-09

**Authors:** Christine Zhiwen Hu, Jaswinder K. Sethi, Thilo Hagen

**Affiliations:** ^1^Department of Biochemistry, Yong Loo Lin School of Medicine, National University of Singapore, Singapore 117597, Singapore; ^2^Institute of Metabolic Science, Metabolic Research Laboratories, and Department of Clinical Biochemistry, University of Cambridge, Addenbrooke's Hospital, Cambridge CB20QQ, UK

## Abstract

*Background*. SOCS proteins are known to negatively regulate insulin signaling by inhibiting insulin receptor substrate-1 (IRS1). IRS1 has been reported to be a substrate for ubiquitin-dependent proteasomal degradation. Given that SOCS proteins can function as substrate receptor subunits of Cullin-5 E3 ubiquitin ligases, we examined whether Cullin-5 dependent ubiquitination is involved in the regulation of basal IRS1 protein stability and signal-induced IRS1 degradation. *Findings*. Our results indicate that basal IRS1 stability varies between cell types. However, the Cullin-5 E3 ligase does not play a major role in mediating IRS1 ubiquitination under basal conditions. Protein kinase C activation triggered pronounced IRS1 destabilization. However, this effect was also independent of the function of Cullin-5 E3 ubiquitin ligases. *Conclusions*. In conclusion, SOCS proteins do not exert a negative regulatory effect on IRS1 by functioning as substrate receptors for Cullin-5-based E3 ubiquitin ligases both under basal conditions and when IRS1 degradation is induced by protein kinase C activation.

## 1. Introduction 

Insulin signaling is an important cellular process which regulates glucose uptake and utilization, lipid and protein synthesis as well as transcriptional responses. Binding of insulin to the insulin receptor (IR) leads to autophosphorylation of the tyrosine kinase domain as well as other proteins which interact with the IR tyrosine kinase. One of the major IR substrates is the insulin receptor substrate (IRS) protein, which is able to dock onto the IR. There are six IRS related proteins, including IRS1-4, Gab1, and p62dok. Upon insulin stimulation, these proteins are phosphorylated on tyrosine residues and subsequently act as a multisite docking protein for src homology 2 (SH2) domain proteins, such as p85 phosphatidylinositol 3-kinase (PI3-kinase). Consequently, the activation of these SH2 domain proteins initiates the insulin dependent signaling cascade. One well-characterized downstream pathway is AKT-dependent translocation and activation of glucose transporters. Another is AKT-dependent activation of the mTOR complex 1 (mTORC1) and the downstream serine/threonine kinase p70 S6 kinase 1 (S6K1). Functionally, this pathway is implicated in regulating transcription, autophagy, ribosome biogenesis, and protein stability. S6K1 has also been shown to directly phosphorylate IRS1 and consequently exerts a negative feedback regulation on IRS1.

Another family of SH2 domain containing proteins is the SOCS (Suppressor Of Cytokine Signaling) protein family. These proteins have originally been implicated in the inhibition of the cellular response to cytokine stimulation. However, SOCS proteins are also known to play a role in regulating the insulin signaling pathway. SOCS protein expression in several tissues and cell lines is increased by insulin [1], and the induced SOCS proteins in turn were shown to negatively regulate insulin signaling [2], suggesting that they also mediate negative feedback regulation of the insulin signaling pathway. 

There are eight members in the SOCS protein family, SOCS1 to SOCS7 and (cytokine-inducible SH2 domain-containing protein) CIS [3]. The sequence of all SOCS proteins is similar, with a variable N-terminal domain, a central SH2 domain and a conserved C terminal SOCS box. While the importance of the SH2 domain for the inhibitory action of SOCS proteins is well established, it is currently not clear to what extent and via what mechanisms the SOCS box contributes to the activity. Interestingly, the SOCS box of these proteins is similar to the *α* domain of the pVHL protein [4]. It is well known that pVHL associates with elongin B/C and Cullin-2, forming an E3 ubiquitin ligase complex which facilitates the ubiquitination and subsequent degradation of hypoxia inducible factor-1*α* (HIF-1*α*), an important mediator of cellular oxygen sensing [5]. Indeed, SOCS proteins have been shown to bind to elongin B/C *in vitro* and *in vivo* [6–9]. Therefore, it can be hypothesized that SOCS proteins may also be able to regulate important proteins in the insulin signaling pathway at the level of their protein stability. Additional clues for the possible function of SOCS proteins come from the finding that, similar to pVHL, SOCS proteins contain a Cullin box, which mediates the binding to Cullin proteins. The Cullin box confers specificity for Cullin proteins, as shown by pVHL binding to CUL2 and SOCS protein binding to Cullin-5 [10]. 

Cullins are scaffold proteins for the assembly of Cullin RING domain E3 ubiquitin ligases. There are seven mammalian cullin proteins (Cullin-1 to Cullin-7), which bind to adaptor proteins and substrate receptor subunits via their N-terminus. This substrate receptor module is responsible for recruiting E3 ligase substrates. For instance, Cullin-5 acts as a scaffold protein which recruits the adaptor proteins elongin B/C and different substrate receptors including SOCS proteins. Rbx2 is a RING domain-containing protein which binds to the C-terminus of Cullin-5 and recruits the E2 conjugating enzyme [10], to facilitate the transfer of ubiquitin onto the substrate. Cullin E3 ligase-mediated polyubiquitination subsequently leads to recognition and degradation of the substrate by the 26S proteasome.

Interestingly, it has been reported that SOCS1 and SOCS3 bind to IRS1 and promote the ubiquitination and degradation of the IRS1 protein [11]. Therefore, the aim of this study is to determine whether Cul5 E3 ubiqutin ligases, utilizing SOCS proteins as adaptor proteins, are involved in the basal and signal induced degradation of IRS1.

## 2. Results

### 2.1. Measurement of Basal IRS1 Protein Stability in Different Cell Lines

**Figure 1:**
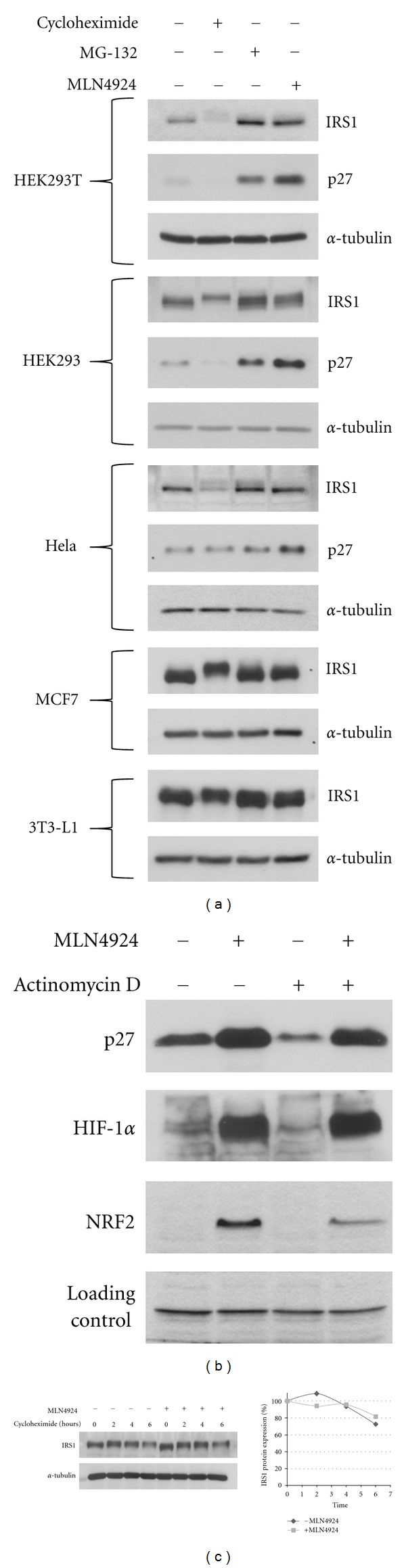
Measurement of basal IRS1 protein stability in different cell lines. (a) Cells were treated for 6 hours with the following drugs prior to cell lysis: cycloheximide (40 *μ*M), MG-132 (20 *μ*M), MLN4924 (3 *μ*M). Subsequently, Western blot analysis was performed. (b) HEK293T cells were pretreated with actinomycin D (5 *μ*g/mL) for 25 min before adding MLN4924 (3 *μ*M) for 4 hours, as indicated. (c) HEK293 cells were preincubated with MLN4924 (1 *μ*M) for 2 hours before adding cycloheximide (40 *μ*M) for the respective time points. Cell lysates were analyzed using Western blotting.

To measure basal rates of IRS1 protein stability, several cell lines were treated with a proteasome inhibitor (MG-132) and an inhibitor of protein synthesis (cycloheximide). Treatment with cycloheximide for 6 hours resulted in a marked reduction in IRS1 protein concentrations in HEK293T, HEK293, and HeLa cells, whereas the effect in MCF7 and 3T3-L1 cells was less pronounced. Similarly, treatment with MG-132 caused a moderate increase in IRS1 protein levels in HEK293T, HEK293, and Hela cell lines but was without effect in MCF7 and 3T3-L1 cell lines. Thus, the IRS1 protein in HEK293T, HEK293 and Hela cells is less stable than in MCF7 and 3T3-L1 cells (Figure 1(a)).

To address the potential involvement of Cullin E3 ligases in regulating basal IRS1 stability, we used the Nedd8 E1 activating enzyme (NAE) inhibitor MLN4924 which inhibits all members of the Cullin E3 ligase family [12, 13]. Cullin E3 ligases require the modification of the cullin protein with the ubiquitin-like protein Nedd8 for their activity. Treatment of cells with MLN4924 is known to result in rapid cullin deneddylation and hence Cullin E3 ligase inhibition. The inhibitory effect of MLN4924 on the Cullin E3 ligase family was confirmed by the marked increase in the protein concentration of p27, HIF-1*α*, and Nrf2, which are bona fide substrates for Cul1, Cul2, and Cul3 E3 ligases, respectively [14], upon treatment with MLN4924 (Figure 1(b)). As expected, stabilization of these Cullin E3 ligase substrates was not affected by treatment with the transcription inhibitor Actinomycin D (Figure 1(b)).

Upon treatment with MLN4924, the IRS1 protein concentration was observed to increase only in HEK293T cells but not in the other cell lines. We also measured the protein abundance of IRS1 in HEK293 cells in the presence or absence of MLN4924 upon inhibition of protein synthesis with cycloheximide. However, no significant differences in the protein abundance of IRS1 was observed over the time course, supported by densitometry measurements (Figure 1(c)). Even in HEK293T cells, where MLN4924 treatment increased the IRS1 protein to similar levels compared to MG-132 (Figure 1(a)), the contribution of Cullin E3 ligases to IRS1 protein stability is likely to be only partial. Thus, in comparison, MLN4924 increased the protein level of the well characterized Cul1 E3 ligase substrate p27, to even higher levels than MG-132 (Figure 1(a)).

**Figure 2:**
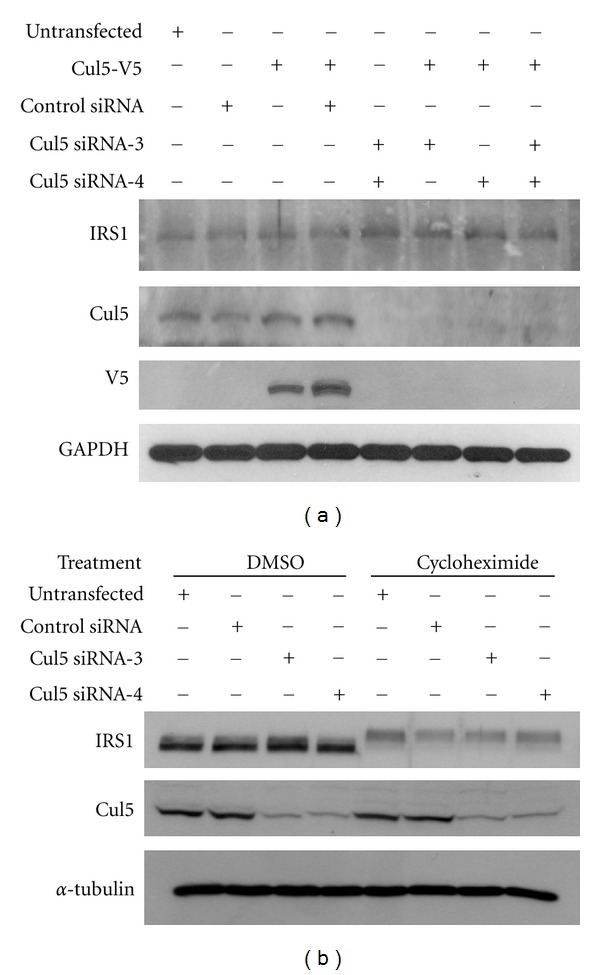
Effect of Cullin-5 knockdown on basal IRS1 protein concentrations. (a) Negative control siRNA (NC) or two different Cullin-5 siRNAs were transfected into HEK293 cells, followed by transfection of Cul5-V5 after 24 hours. After further 24 hours, the cells were lysed with detergent lysis buffer and analyzed by Western blotting. (b) HEK293 cells were transfected with negative control or the respective Cul5 siRNA. After three days, the cells were treated with cycloheximide (40 *μ*M) for 6 hours, lysed, and analyzed by Western blotting.

To investigate the involvement of Cul5 in basal IRS1 degradation, we tested whether overexpression or siRNA-mediated silencing of this Cullin homologue in HEK293 cells affects IRS1 protein expression. As shown in Figure 2(a), IRS1 protein levels were not significantly altered upon overexpression or knockdown of Cul5. The above results were confirmed by using cycloheximide treatment in cells with Cul5 knockdown. Upon inhibition of new protein synthesis by cycloheximide, the turnover of IRS1 was very similar in untransfected cells and in cells transfected with control or Cul5 siRNA (Figure 2(b)), strongly suggesting that degradation of basal IRS1 is not mediated by Cul5. Taken together, the results suggest that Cullin E3 ligases do not play a major role in regulating basal IRS1 protein stability in these cells. Given that it has been reported that different physiological signals are able to induce IRS1 degradation, we investigated in further experiments whether Cullin E3 ligase-dependent ubiquitination is involved in these regulatory mechanisms.

### 2.2. Effect of mTOR/S6K1 and TPA on IRS1 Protein Stability

**Figure 3:**
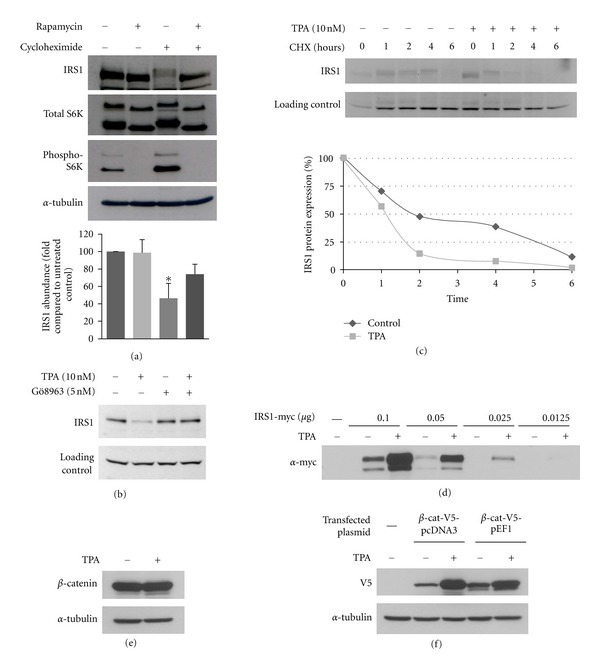
Effect of mTOR/S6K1 and TPA on IRS1 protein stability. (a) HEK293T cells were treated with rapamycin (20 nM) and cycloheximide (40 *μ*M), as indicated, for 6 h followed by cell lysis and Western blot analysis. IRS1 protein abundance was determined by densitometry analysis indicated above the band as fold compared to the untreated control. All forms of IRS1 (shifted and not shifted) were included in the densitometry analysis. (b) HEK293T cells were preincubated with Gö8963 (5 nM) for one hour before 24 hour incubation with TPA (10 nM), as indicated. (c) HEK293 cells were pretreated with 10 nM TPA for 1 hour before cycloheximide chase was conducted using the indicated time points. Immunoblot analysis of lysates was then carried out. (d) HEK293T cells were transfected with the specified amounts of IRS1. After the cells reached subconfluence, TPA (10 nM) was added to the medium for 24 hours before cell lysis. (e) HEK293 cells were treated with TPA (10 nM) for 24 hours followed by Western blot analysis of cell lysates for endogenous *β*-catenin. (f) HEK293 cells were transfected with 0.5 *μ*g of the indicated *β*-catenin plasmids. After two days, TPA (10 nM) was added for 24 hours followed by Western blot analysis of cell lysates with the indicated antibodies.

It is well known that chronic treatment with growth factors induces a negative feedback regulation on IRS1 via the mTOR/S6K1 pathway, leading to S6K1-dependent phosphorylation of IRS1. We therefore tested the effect of inhibiting basal mTOR activity in the presence of serum on IRS1 protein concentrations. As expected, upon addition of the mTOR inhibitor rapamycin, phosphorylation of the mTORC1 downstream target p70 S6K was completely prevented (Figure 3(a)). Rapamycin treatment also resulted in an increased mobility of IRS1, indicating that S6K1 contributes to the basal phosphorylation of IRS1. However based on densitometry results from three independent experiments, rapamycin had no effect on IRS1 protein steady-state levels. This result suggests that mTOR/S6K1 does not regulate IRS1 basal protein stability. As previously reported, cycloheximide treatment caused a robust activation of mTORC1 as detected by an increase in S6K activity [15]. Cycloheximide was also found to lower the mobility of IRS1 compared to the untreated control indicating that the phosphorylation of IRS1 was induced. Upon coincubation of cycloheximide with rapamycin, phosphorylation of S6K was fully inhibited and likewise the phosphorylation of IRS1 was inhibited (Figure 3(a), lane 4). The IRS1 protein steady-state level after 6 hours of cycloheximide was decreased to 46% compared to the control. Interestingly, inhibition of mTOR partially prevented the decrease in IRS1 protein (decrease to 74% compared to control). In conclusion, our results suggest that under basal conditions in the presence of growth factors, mTOR/S6K is not involved in regulating IRS1 protein stability. In contrast, upon activation to higher levels, mTOR/S6K may be involved in inducing IRS1 degradation.

**Figure 4:**
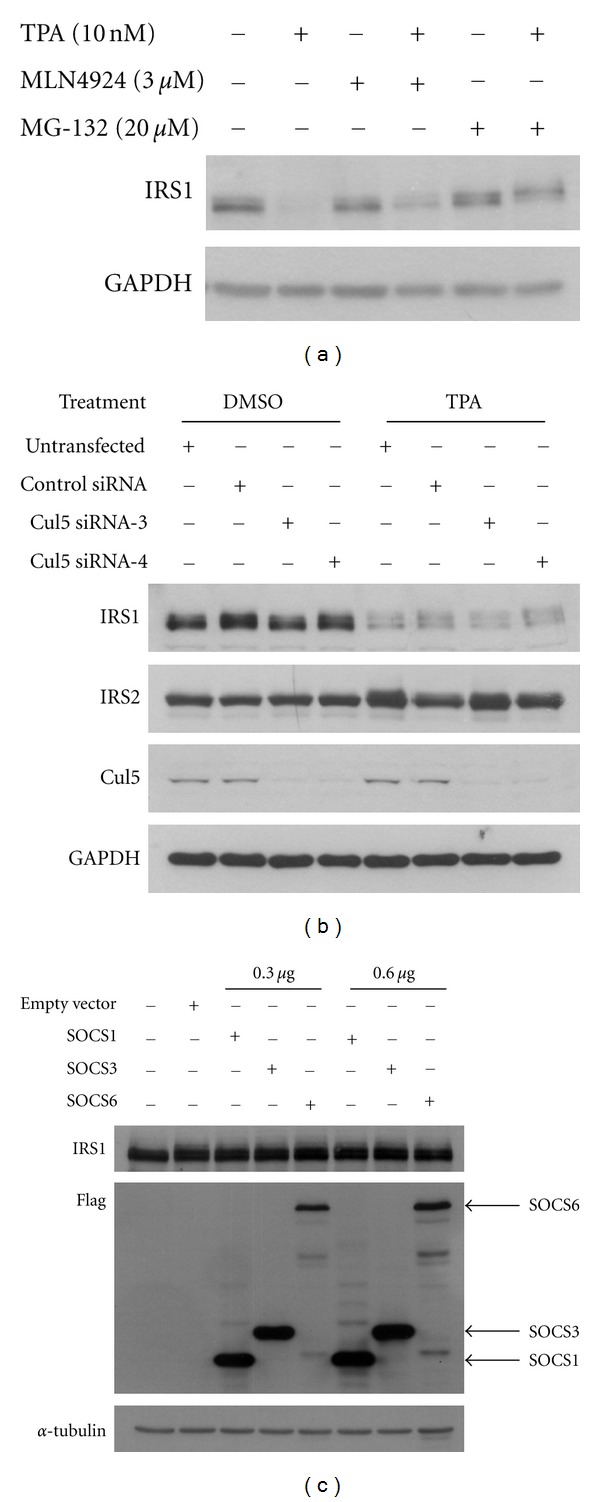
Role of the Cullin-5 E3 ligase in TPA-induced IRS1 degradation. (a) HEK293 cells were treated TPA (10 nM) for the last 24 hours, with MLN4924 (3 *μ*M) (24 hours) and MG-132 (20 *μ*M) (6 hours), as indicated. (b) HEK293 cells were transfected with negative control or Cullin-5 siRNA. After two days, TPA (10 nM) was added for 24 hours. Subsequently, the cells were lysed for Western blot analysis. (c) HEK293T cells were transfected with the respective amounts of SOCS plasmid as indicated. Immunoblot analysis of lysates was subsequently carried out.

Phorbol ester (TPA) has been reported to negatively regulate IRS1 protein concentrations via PKC activation. With this in mind, we used TPA to activate PKC in HEK293T cells. As expected, IRS1 protein concentrations markedly decreased upon addition of TPA, and this effect was reversed in the presence of the PKC inhibitor Gö8963 (Figure 3(b)). Downregulation of IRS1 protein by TPA was prevented in the presence of the proteasome inhibitor MG-132 (see Figure 4(a)), indicating that PKC activation induces IRS1 degradation. To confirm that the TPA-induced IRS1 downregulation is due to an effect on protein stability, a cycloheximide chase experiment was conducted in the presence or absence of TPA. As shown in Figure 3(c), the degradation rate of IRS1 under TPA treatment is faster than in the control.

We also investigated the effect of TPA on transfected IRS1. Unlike the TPA-induced degradation of endogenous IRS1, we observed that transfected IRS1 protein showed a consistent increase in abundance upon treatment with TPA (Figure 3(d)). To determine whether this effect was due to a nonspecific effect of TPA on the CMV promoter of the transfected plasmid, experiments were performed to determine the effect of TPA on endogenous and transfected *β*-catenin expression. As expected, the endogenous *β*-catenin protein levels remained unchanged upon treatment with TPA (Figure 3(e)). When *β*-catenin expression plasmids with either a CMV or a EF2 promoter were transfected into cells, a marked upregulation of transfected *β*-catenin was observed with both promoters (Figure 3(f)), albeit the CMV promoter showed a greater effect compared to the EF2 promoter. This confirmed that TPA has a nonspecific effect on the promoters of plasmids and therefore, in subsequent experiments we only studied the effects of TPA on endogenous IRS1 protein.

### 2.3. Cul5 Is Not Involved in TPA-Induced IRS1 Degradation

To determine whether the TPA-induced decrease in endogenous IRS1 is due to Cullin-dependent proteasomal degradation, the NAE inhibitor MLN4924 was added to TPA treated cells. As shown in Figure 4(a), the proteasome inhibitor MG-132 prevented the degradation of IRS1 almost completely, whereas MLN4924 (lane 5) only partially rescued the IRS1 protein expression. Therefore, these results show that TPA-induced IRS1 degradation is only partially dependent on Cullin E3 ligases.

Given the suggested role of SOCS proteins acting as Cul5 substrate receptors in regulating IRS1 stability, we sought to determine the role of the Cul5-based Cullin E3 ligase in TPA-induced IRS1 degradation. To this end, control and Cul5 siRNA duplexes were transfected into cells and 2 days after transfection, TPA was added for 24 hours. Consistent with results shown above, Western blot analysis revealed that basal IRS1 protein levels remained unchanged upon Cul5 siRNA-mediated silencing (Figure 4(b)). Importantly, Cul5 knockdown was without effect on the TPA-induced degradation of IRS1, despite a marked reduction in Cul5 protein levels. Furthermore, TPA treatment did not have an effect on IRS2 protein levels.

Finally, to directly test whether SOCS proteins are able to promote the degradation of IRS1, overexpression plasmids for SOCS1, SOCS3, and SOCS6, which have been implicated in inhibiting IRS1 [11], were generated and expressed in HEK293T cells. As shown in Figure 4(c), robust overexpression levels of SOCS1, SOCS3, and SOCS6 did not induce the degradation of IRS1 protein. Coexpression of human insulin receptor and SOCS proteins in both HEK293 and HEK293T cells was also without significant effect on IRS1 protein levels (data not shown). In conclusion, our data suggest that SOCS proteins do not exert a negative regulatory effect on IRS1 by functioning as substrate receptors for the Cul5-based Cullin E3 ligase, both under basal conditions and when IRS1 degradation is induced by treatment with TPA.

### 2.4. Discussion

SOCS proteins have been shown to negatively regulate insulin signalling by inhibiting IRS1. The IRS1 protein has been reported to be a substrate for ubiquitin-dependent proteasomal degradation [16, 17]. When measuring basal protein turnover rates of IRS1 in different cell lines, we found that the protein was more unstable in HEK293, HEK293T, and HeLa cells compared to MCF7 and 3T3-L1 cells. The different basal degradation rates may be due to differences in posttranslational modifications in the IRS1 protein that are important to induce its ubiquitination and degradation or due to differences in the expression levels or activities of E3 ubiquitin ligases involved in IRS1 ubiquitination. SOCS1 and SOCS3 have been reported to induce IRS1 degradation [11]. Given that SOCS proteins can function as substrate receptor subunits for Cullin-5 E3 ubiquitin ligases, it is possible that they function to recruit IRS1 for ubiquitination. However, our results using Cullin-5 knockdown and overexpression of SOCS proteins argue against this possibility. Because the original study by Rui et al. [11] used a cellular system where insulin receptor was overexpressed, we also measured the IRS1 protein stability in cells transfected with a human insulin receptor plasmid. However, overexpression of insulin receptor alone or with various SOCS proteins did not significantly affect IRS1 degradation. Therefore, it is likely that other cullin and non-cullin based E3 ligases exist which can mediate basal IRS1 protein turnover. However, we cannot rule out that there are cell-type specific IRS1 ubiquitination mechanisms that involve Cullin-5 or other Cullin-based E3 ubiquitin ligases.

The phosphorylation of IRS1 is likely to be important for the regulation of its protein stability. For instance, mTOR/S6K exerts a negative feedback on insulin signalling via IRS1 phosphorylation. This negative feedback could be due to inhibition of IRS1 activity and/or induction of IRS1 degradation. Our results suggest that mTOR/S6K might not be involved IRS1 degradation under basal conditions in the presence of serum. However when increased mTOR/S6K signalling occurs, for example, in the presence of cycloheximide, IRS1 protein becomes more unstable.

The phorbol ester TPA is a well known activator of protein kinase C *α* (PKC*α*) [18]. PKC*α* phosphorylates a wide range of substrates, including IRS proteins. It has been reported that PKC*α*-mediated phosphorylation causes inhibition of IRS1 activity [19]. In our studies we found that TPA decreased the IRS1 protein half-life. Furthermore, the PKC inhibitor Gö8963 was able to restore the IRS1 protein level in the presence of TPA. In order to characterize the molecular basis of TPA-induced IRS1 protein degradation, that is, to identify involved phosphorylation sites and IRS1 ubiquitin modification, we intended to use transfected versions of IRS1. However, our experimental results indicated that the transfected IRS1 protein was consistently upregulated upon treatment with TPA, and this effect is likely due to a nonspecific effect of TPA on the promoter of the transfected plasmid.

Since TPA induced a robust degradation of the endogenous IRS1 protein, the role of Cul5 mediated ubiquitination was determined using NAE inhibitor MLN4924 and siRNA-mediated gene silencing. Cul5 knockdown did not restore IRS1 protein levels in the presence of TPA, suggesting that Cullin-5 might not be the Cullin E3 ligase responsible for the degradation of IRS1. We observed that MLN4924 partially inhibited TPA-induced IRS1 degradation. However, it appears unlikely that TPA induces IRS1 degradation via more than one cellular E3 ubiquitin ligase. Therefore, the observed effect of MLN4924 may be due to inhibition of basal IRS1 protein turnover, while TPA induced degradation is likely mediated via a different, non-cullin based cellular E3 ubiquitin ligase.

In conclusion, our results indicate that the inhibition of IRS1 by SOCS proteins is not primarily mediated via their function as substrate receptor subunits of Cullin-5 based E3 ubiquitin ligases. Thus, other non-Cullin-5 based cellular E3 ligases are likely to be responsible for basal and signal induced IRS1 protein degradation.

## 3. Materials and Methods

### 3.1. Cell Culture

HEK293, HEK293T, HeLa, and 3T3-L1 cells were grown in DMEM medium and MCF7 cells in RPMI medium. All media were supplemented with penicillium/streptomycin, 10% fetal bovine serum (FBS), and L-glutamine. 

### 3.2. Immunoblotting

Cells were washed with ice-cold 1X PBS and lysed in lysis buffer with 0.1%  *β*-mercaptoethanol. Lysates were precleared using centrifugation and equal amounts of proteins were loaded using the Bradford protein assay. The following antibodies were used: monoclonal anti-glyceraldehyde-3-phosphate dehydrogenase (G8140-04; U.S. Biological), monoclonal anti-tubulin (236-10501; Molecular Probes), monoclonal anti-myc (2276; Cell Signaling), polyclonal anti-IRS1 (sc-7200; Santa Cruz Biotechnology), polyclonal anti-IRS2 (06-506; Upstate), monoclonal anti-p27 (610241; BD Biosciences), polyclonal anti-Nrf2 (sc722; Santa Cruz Biotechnology), monoclonal anti-V5 (Serotec), polyclonal anti-p70 S6 Kinase (9202; Cell Signaling), monoclonal anti-phospho-p70 S6 Kinase (Thr389) (9234; Cell Signaling), monoclonal anti-HIF-1*α* (610959; BD Pharmingen), and polyclonal anti-Cul5 (sc-13014; Santa Cruz Biotechnology). Western blots shown are representative of at least two independent experiments.

### 3.3. siRNA-Mediated Gene Silencing

siRNA transfection was carried out using RNAi Max lipofectamine (Invitrogen) according to the manufacturer's instruction. Cells were lysed three days after siRNA knockdown for Western blot analysis, as described above. Cullin-5 siRNAs were obtained from Integrated DNA Technologies (HSC.RNAI.N3478.10.3 and HSC.RNAI.N3478.10.4).

### 3.4. Plasmid Constructs and Transfection of Cells

The plasmids pcDNA3.1-Myc-his-mIRS1 and pcDNA3.1-Myc-his-mIRS2 were generated as previously reported [19]. To generate the *β*-catenin plasmids, *β*-catenin coding sequence including a C-terminal V5 tag, was inserted into KpnI and XbaI sites of two vectors, pcDNA3 and pEF1. The human SOCS3 and SOCS6 cDNA clone was purchased from Geneservice (I.M.A.G.E ID 30333577 and 3917519). To generate C-terminally FLAG tagged SOCS3 and SOCS6, clones was PCR amplified and inserted into pcDNA3. The human SOCS1 was PCR amplified from cDNA and inserted into modified pcDNA3.1 with N-terminal FLAG tag. Sub-confluent cells were transfected using Genejuice (Novagen) according to the manufacturer's instructions. 

## References

[1] Banks AS, Li J, McKeag L (2005). Deletion of SOCS7 leads to enhanced insulin action and enlarged islets of Langerhans. *Journal of Clinical Investigation*.

[2] Emanuelli B, Peraldi P, Filloux C, Sawka-Verhelle D, Hilton D, Van Obberghen E (2000). SOCS-3 is an insulin-induced negative regulator of insulin signaling. *The Journal of Biological Chemistry*.

[3] Hilton DJ, Richardson RT, Alexander WS (1998). Twenty proteins containing a C-terminal SOCS box form five structural classes. *Proceedings of the National Academy of Sciences of the United States of America*.

[4] Babon JJ, Sabo JK, Zhang JG, Nicola NA, Norton RS (2009). The SOCS box encodes a hierarchy of affinities for Cullin5: implications for ubiquitin ligase formation and cytokine signalling suppression. *Journal of Molecular Biology*.

[5] Ohh M, Park CW, Ivan M (2000). Ubiquitination of hypoxia-inducible factor requires direct binding to the *β*-domain of the von Hippel-Lindau protein. *Nature Cell Biology*.

[6] Kibel A, Iliopoulos O, DeCaprio JA, Kaelin WG (1995). Binding of the von Hippel-Lindau tumor suppressor protein to Elongin B and C. *Science*.

[7] Aso T, Haque D, Barstead RJ, Conaway RC, Conaway JW (1996). The inducible elongin A elongation activation domain: structure, function and interaction with the elongin BC complex. *EMBO Journal*.

[8] Kamura T, Sato S, Haque D (1998). The Elongin BC complex interacts with the conserved SOCS-box motif present in members of the SOCS, ras, WD-40 repeat, and ankyrin repeat families. *Genes and Development*.

[9] Zhang JG, Farley A, Nicholson SE (1999). The conserved SOCS box motif in suppressors of cytokine signaling binds to elongins B and C and may couple bound proteins to proteasomal degradation. *Proceedings of the National Academy of Sciences of the United States of America*.

[10] Kamura T, Maenaka K, Kotoshiba S (2004). VHL-box and SOCS-box domains determine binding specificity for Cul2-Rbx1 and Cul5-Rbx2 modules of ubiquitin ligases. *Genes and Development*.

[11] Rui L, Yuan M, Frantz D, Shoelson S, White MF (2002). SOCS-1 and SOCS-3 block insulin signaling by ubiquitin-mediated degradation of IRS1 and IRS2. *The Journal of Biological Chemistry*.

[12] Soucy TA, Smith PG, Milhollen MA (2009). An inhibitor of NEDD8-activating enzyme as a new approach to treat cancer. *Nature*.

[13] Brownell JE, Sintchak MD, Gavin JM (2010). Substrate-assisted inhibition of ubiquitin-like protein-activating enzymes: the NEDD8 E1 inhibitor MLN4924 forms a NEDD8-AMP mimetic in situ. *Molecular Cell*.

[14] Lee J, Zhou P (2010). Cullins and cancer. *Genes and Cancer*.

[15] Finlay D, Ruiz-Alcaraz AJ, Lipina C, Perrier S, Sutherland CD (2006). A temporal switch in the insulin-signalling pathway that regulates hepatic IGF-binding protein-1 gene expression. *Journal of Molecular Endocrinology*.

[16] Zhande R, Mitchell JJ, Wu J, Sun XJ (2002). Molecular mechanism of insulin-induced degradation of insulin receptor substrate 1. *Molecular and Cellular Biology*.

[17] Briaud I, Dickson LM, Lingohr MK, McCuaig JF, Lawrence JC, Rhodes CJ (2005). Insulin receptor substrate-2 proteasomal degradation mediated by a mammalian target of rapamycin (mTOR)-induced negative feedback down-regulates protein kinase B-mediated signaling pathway in *β*-cells. *The Journal of Biological Chemistry*.

[18] Bouché M, Zappelli F, Polimeni M (1995). Rapid activation and down-regulation of protein kinase C *α* in 12-O-tetradecanoylphorbol-13-acetate-induced differentiation of human rhabdomyosarcoma cells. *Cell Growth and Differentiation*.

[19] Nawaratne R, Gray A, Jørgensen CH, Downes CP, Siddle K, Sethi JK (2006). Regulation of insulin receptor substrate 1 pleckstrin homology domain by protein kinase C: role of serine 24 phosphorylation. *Molecular Endocrinology*.

